# Evaluation of Properties of Mineral Trioxide Aggregate with Methyl Cellulose as Liquid

**Published:** 2017-01

**Authors:** Omid Dianat, Mandana Naseri, Seyedeh Farnaz Tabatabaei

**Affiliations:** 1 Associate Professor, Department of Endodontics, Dental School, Shahid Beheshti University of Medical Sciences, Tehran, Iran; 2 Postgraduate Student of Restorative and Cosmetic Dentistry, Department of Restorative and Cosmetic Dentistry, Islamic Azad University, Tehran, Iran

**Keywords:** Compressive Strength, Mineral Trioxide Aggregate, Tensile Strength

## Abstract

**Objectives:**

Mineral trioxide aggregate (MTA) is extensively used in endodontics. However, MTA is difficult to handle because of its granular consistency, low mechanical properties and initial looseness. The objective of this study was to assess the compressive strength (CS), diametral tensile strength (DTS), and pH of set MTA using methyl cellulose as liquid.

**Materials and Methods:**

White ProRoot MTA was used as the control group; modified MTA cement was prepared by mixing Portland cement, bismuth oxide and calcium sulfate (75%, 20% and 5%, respectively) as the experiment group. Methyl cellulose was used as hydrating liquid and compared with distilled water. The data were analyzed by two-way ANOVA.

**Results:**

The pH values of modified MTA cement set using deionized water and methyl cellulose were slightly, but not significantly, different (P>0.05). The DTS and CS tests for modified MTA cement hydrated with methyl cellulose showed a significant difference at one day and one week (P<0.05).

**Conclusions:**

The results suggest that using methyl cellulose as the hydrating liquid enhances some mechanical properties but does not compromise pH of white ProRoot MTA.

## INTRODUCTION

Mineral trioxide aggregate (MTA), a powder mixture consisting of hydrophilic particles including Portland cement clinker, bismuth oxide and gypsum, is extensively used as a root canal sealer and for pulp capping, apexification, and root end filling [1,2]. After a hydration reaction, the byproducts of insoluble calcium silicate hydrate and alkaline calcium hydroxide offer MTA cement unique stability and sealing ability, antibacterial properties, biocompatibility, and potential to enhance hard tissue regeneration [3]. However, the handling properties of MTA complicate its use, for example, in apical surgeries with limited access; MTA is difficult to handle, because of its granular consistency, low strength and initial looseness, specifically as a pulp capping material [4,5]. A biocompatible cement hydration liquid, comprising of 3% methyl cellulose, 1.5% polyethylene glycol and 0.5% sodium fluoride is claimed to extensively improve the mechanical properties of MTA [6]. Polyethylene glycol is an anti-washout, anti-shrinkage solvent used as a lubricating coating for various surfaces in aqueous and non-aqueous environments [7]. Methyl cellulose is composed of nonionic water-soluble cellulose ether, which has an OH base and is almost like water. The methyl cellulose anti-washout admixture binds water molecules within the cement and increases the cohesive strength of the material, improving its handling and mechanical properties. Methyl cellulose helps control several important properties of a formulation such as rheology, dispersion, water demand and water retention. It has many practical benefits, including high consistency and workability, high standing strength and high yield [7]. Therefore, the present study was designed to examine the compressive strength (CS), diametral tensile strength (DTS) and pH of MTA mixed with methyl cellulose as liquid.

## MATERIALS AND METHODS

The modified MTA cement was prepared by mixing Portland cement (75%), bismuth oxide (20%), and gypsum (5%) in a bench-top planetary ball mill (Retsch PM100; Retsch GmbH, Haan, Germany) for 10 minutes [8]. The MTA cement was individually mixed with distilled water and 3% methyl cellulose. The powder/methyl cellulose ratio of 1/1 by weight was tried first. The powder and methyl cellulose were weighed on a glass slab at room temperature. The powder was then divided into four equal portions on the glass slab. Mixing was started by mixing the first portion of the powder with methyl cellulose. The process was continued to gain the desired consistency. The ultimate amount of powder that can be added to methyl cellulose to obtain a homogenous consistency was used. Thus, the ratio of powder to methyl cellulose needed for mixing was determined to be 4:1 by weight. White ProRoot MTA (Dentsply, Tulsa Dental, Tulsa, OK) mixed with distilled water according to the manufacturer’s instructions, was used as the control group.

### Compressive strength and DTS:

The CS of the test materials was determined using the ISO 9917-1 method [9]. Each material was mixed and placed in a split stainless steel mold (4.0mm inner diameter and 6.0mm height). No later than 120 seconds after mixing, the mixture was transferred to an incubator at 37°C and 100% relative humidity. Samples were removed from the molds and checked visually for any voids or chipped edges. All defective samples were discarded. Totally, 120 acceptable samples were prepared of each cement: 10 samples for each test at predetermined time points. All samples were incubated at 37°C in fully saturated humidity for three and seven days. The CS and DTS were then measured by a universal testing machine (Model GB/4302; Instron Corporation, High Wycombe, Bucks, United Kingdom) at a crosshead speed of 0.5 mm/minute. The maximum load needed for fracture of each sample was measured. The CS was calculated in megapascals (MPa) according to the formula: CS ¼ 4P=PD2, where P is the maximum load applied in Newtons (N), and D is the mean diameter of the sample in millimeters. The DTS of cylindrical samples was calculated from the following formula: DTS= 2P/Pbw, where P is the peak load (N), b is the diameter (mm), and w is the length (mm) of the cylinder [8–12].

### The pH measurement:

For the pH test, 10 polyethylene tubes measuring 3mm in diameter and 1mm in height were filled with freshly prepared samples of each material, sealed in flasks containing 10mL of distilled water, and stored at 37°C. The pH was measured at the end of mixing. After three hours of immersion, the tubes were carefully removed and placed into new flasks with an equal amount of fresh distilled water, and this procedure was repeated after every predetermined period of 48 and 72 hours and seven days [13–16]. The pH of the solutions was analyzed at each time point using a previously calibrated digital pH meter (Seven Easy; Mettler Toledo, Schwerzenbach, Switzerland).

### Statistical analysis:

Repeated measures ANOVA was used to examine the differences in pH. The data from the CS and DTS tests were analyzed by two-way ANOVA. In all cases, the results were considered statistically significant at P<0.05.

## RESULTS

### Compressive strength and DTS:

Table 1 shows the strength values of the three cement groups. The compressive strength values of White MTA and modified MTA cement mixed with distilled water did not significantly differ after three days (P=0.873) and one week (P=0.239). The DTS of White MTA and modified MTA cement mixed with distilled water did not significantly differ after three days (P=0.889) and one week (P=0.255). The CS and DTS of all admixtures increased significantly during seven days (P<0.001). The strength of modified MTA cement mixed with methyl cellulose was significantly higher than that of modified MTA cement mixed with distilled water and White MTA at all time points (P<0.001).

**Table 1:** Mean and standard deviation of strength for different materials at different time points

**Cement type**	**Compressive strength (3h, MPa)**	**Compressive strength (7D, MPa)**	**Tensile strength (3h, MPa)**	**Tensile strength (7D, MPa)**
MTA+DW	14.49±1.26	20.57±1.67	1.92±0.21	6.92±0.31
MTA+MC	20.17±2.90	29.98±2.67	2.24±0.18	10.01±0.36
WMTA+DW	14.97±2.08	22.06±1.41	2.06±0.26	7.21±0.52

WMT A: White mineral trioxide aggregate; DW: Distilled water; MC: Methyl cellulose; H: Hours; MPa: Megapascals; D: Days

### The pH variation:

The results of pH measurements are presented in Table 2. The pH values of White MTA and modified MTA cement mixed with distilled water did not significantly differ at all time points (P=0.752). The pH values of all admixtures increased significantly during the seven-day period (P<0.001). The pH values of modified MTA cement mixed with methyl cellulose were slightly, but not significantly, lower than those of modified MTA cement mixed with distilled water and White MTA at all time points (P=0.752).

**Table 2:** Mean and standard deviation of pH values for different materials at different time points

**Cement type**	**Freshly mixed**	**3h**	**48h**	**72h**	**7D**
MTA+DW	11.07±0.21	11.48±0.25	11.53±0.25	11.59±0.25	11.89±0.27
MTA+MC	10.93±0.20	11.44±0.23	11.48±0.22	11.53±0.23	11.84±0.24
WMTA+DW	11.21±0.24	11.67±0.21	11.51±0.23	12.02±0.19	11.97±0.29

WMT A: White mineral trioxide aggregate; DW: Distilled water; MC: Methyl cellulose; H: Hours; D: Days

## DISCUSSION

White MTA primarily consists of inorganic particles such as tricalcium silicate, dicalcium silicate, tricalcium aluminate, bismuth oxide, and gypsum. When cement and water are mixed, the hydration reactions are mostly exothermic and are governed by dissolution-precipitation processes. Consequently, the influence of the liquid phase, such as water, on the dissolution of inorganic particles, the nucleation growth processes of calcium silicate hydrate, and the precipitation of calcium hydroxide are dominant factors affecting the hydration rate of MTA. Hydrated cement creates a calcium silicate hydrate-based colloidal gel that solidifies to form an impermeable barrier with high alkalinity [4,17]. The greater mechanical properties of MTA are considered an important feature when this cement is used as pulp capping or as a coronal restorative material where it is subjected to occlusal loads. Typical test parameters to assess mechanical properties of hydrated cement include compressive and tensile strengths. However, when MTA is used as a root-end filling material, where minimal forces are applied, its reduced strength will not be a major drawback [18]. The test method of the splitting tensile strength, also known as DTS, is commonly used when conventional tensile testing is difficult to perform because of the brittle nature of the test material. The flexural strength of MTA is significantly affected by the setting conditions [19] and condensation pressure [20]. Ji et al, [8] showed that DTS values of the modified MTA cement with distilled water was significantly higher than the values obtained using calcium lactate gluconate liquid after one day. However, at 21 days, distilled water and calcium lactate gluconate liquid systems did not significantly differ. The MTA with 10% CaCl2, 20% CaF, and 15% Na2HPO4 exhibited significantly lower compressive strength compared to the use of distilled water at seven days [21]. Different results were observed in our short-term study; modified MTA cement mixed with methyl cellulose showed higher strength values on days three and seven; because methyl cellulose is claimed to provide high consistency, high standing strength and high yield [7]. However, when mixing MTA powder, substitution of 0.12% chlorhexidine for water significantly increased the compressive strength at one week [22]. When MTA powder is mixed with water, calcium hydroxide and calcium silicate hydrate are formed initially and eventually transform into a poorly crystallized and porous solid gel [23]. The ratio of calcium silicate decreases because of the formation of calcium precipitate. The precipitated calcium produces calcium hydroxide, which causes high alkalinity of MTA after hydration [24]. This suggests that MTA mixed with hydration accelerators has lower pH than MTA mixed with distilled water. The biological properties of MTA (e.g. the ability to induce changes in cellular activity of osteoblasts) have been attributed to its alkalinity [25]. However, the pH of the modified MTA cement mixed with methyl cellulose was not significantly different from that of white MTA and modified MTA cement mixed with distilled water at all time points. This result indicates that MTA has a strong potential for releasing hydroxyl ions [24]. Negm et al, [26] evaluated the pH of an experimental sealer based on Portland cement compared with MTA and found that by addition of calcium hydroxide pH values were not significantly affected, which was also observed in the present study. Prasad et al, [21] showed that the pH of MTA mixed with CaCl2 was lower than that of MTA; however, when CaF and Na2HPO4 were added to MTA, pH value obtained was higher than that of MTA. The pH values observed in this study were smaller than those obtained by Parirokh and Torabinejad [1]. This is probably because of the use of tubes in this experiment, which limited contact to the surrounding water. Another factor to be considered is that the material was placed in a new flask with fresh distilled water at each evaluation time point.

## CONCLUSION

According to the findings of this study, using methyl cellulose solution as an additive can improve the mechanical properties of hydrated MTA without comprising its pH. The expression of mineralization markers may need to be further investigated to verify the usefulness of this modified mixture in clinical applications.
